# Correlates of Chlamydia and Gonorrhea Infection among Female Sex Workers: The Untold Story of Jiangsu, China

**DOI:** 10.1371/journal.pone.0085985

**Published:** 2014-01-15

**Authors:** Weiming Tang, Jicheng Pan, Ning Jiang, Hai-Yang Hu, Tanmay Mahapatra, Yue-Ping Yin, Sanchita Mahapatra, Xiao-Liang Wang, Xiang-Sheng Chen, Nan Lin, Xun Zhang, Xi-Ping Huan, Hai-Tao Yang, Geng-Feng Fu

**Affiliations:** 1 Department of Epidemiology, School of Public Health, University of California, Los Angeles, California, United States of America; 2 Jiangsu Provincial Central for Disease Prevention and Control, Nanjing, Jiangsu, China; 3 Jiangsu Province Geriatric Institute, Nanjing, Jiangsu, China; 4 National Center for STD Control, China CDC, Nanjing, Jiangsu, China; 5 College of Ocean & Earth Science, Xiamen University, Xiamen, China; 6 Department of Molecular Pharmacology and Physiology, MCM, University of South Florida, Tampa, Florida, United States of America; UCL Institute of Child Health, University College London, United Kingdom

## Abstract

**Objective(s):**

To estimate the prevalence of sexually transmitted infections (STIs) among female sex workers (FSWs) in the Jiangsu Province, China and measure the association of Chlamydia trachomatis (CT) and Neisseria gonorrhoeae (NG) infections with their potential correlates.

**Design:**

A cross-sectional study on a representative sample of FSWs in Yangzhou and Changzhou cities of Jiangsu was conducted.

**Methods:**

185 sex-work venues in Yangzhou and 174 in Changzhou were selected by stratified random sampling. 2972 FSWs (1108 in Yangzhou and 1864 in Changzhou), aged 15 years or more, who agreed to participate and provided blood sample for HIV and syphilis testing were interviewed in these venues. Cervical specimens from 849 randomly chosen participants were then tested for CT and NG.

**Results:**

Proportions of young, school-educated, currently married FSWs who were living alone, migrated from other provinces and engaged in unprotected vaginal intercourse in past 3 months (UVI) were relatively high. Prevalence of HIV, syphilis, CT and NG were 0.20%, 4.88%, 14.61% and 5.42% respectively. Younger age, living alone or with persons other than partners/family members, engaging in UVI and having other STIs seemed to be associated with higher risk of CT or NG infection. Being divorced/widowed and working in middle/low-level venues were identified as additional risk factors for NG.

**Conclusions:**

Based on a representative sample, this initial effort to identify the correlates of CT/NG infections among FSWs of Jiangsu revealed that focused interventions targeting high-risk FSWs are urgently required for controlling STI epidemics in Yangzhou and Changzhou where substantial number of STI cases were identified.

## Introduction

At the end of the year 2011, the estimated number of People Living With HIV/AIDS (PLWHA) was about 780,000 in China with a national HIV prevalence of 0.058% [1]. Among these HIV patients, 46.5% (2011 estimates, increased from 44.3% in 2009) were found to be infected through heterosexual route [1]. Since 2005, heterosexual contact had become the dominant HIV transmission route in this country [2]. Female Sex Workers (FSWs) played an important role in this HIV epidemic [3] owing to their increased propensity of having unprotected sex and concurrent partners [4]. In China, the number of females involved in this trade had reached 10 million in 2003 which generated serious public health concerns in terms of magnitude of the impact of their role in HIV epidemic [4]. According to the recent reports from sentinel surveillance sites, HIV prevalence among FSWs exceeded 1% in Yunnan, Xinjiang, Guangxi, Sichuan and Guizhou provinces/autonomous regions during 2011 [1].

Besides HIV/AIDS, public health concerns of this country also include epidemics of Sexually Transmitted Infections (STIs) caused by Treponema pallidum (syphilis), Chlamydia trachomatis (CT) and Neisseria gonorrhoeae (NG) while it has also been well established that the presence of any of these infections potentially increases the risk of acquisition of HIV by manifolds [5]. A report published in 2010 revealed that the expanding syphilis epidemic was very much likely to worsen the HIV scenario in China [6]. While 9% of Chinese males were found to be engaged in commercial sex, the bridging role of FSWs between high-risk and general population seemed to play a significant role in the upsurge of STI epidemic here [7]. A review of studies conducted between 1996–2010 involving FSWs in different regions across the country revealed the range of prevalence of active syphilis being 0.8–12.5% (median = 6.9%), chlamydia being 3.9–58.6% (median = 25.7%) and gonorrhea being 2.0–85.4% (median = 16.4%) [7]. Another study conducted in 8 cities of China between June and September 2009 reported CT and NG prevalence among FSWs to be as high as 5.91% and 17.30% respectively [3].

Like other regions, in the coastal province of Jiangsu in eastern China concentrated epidemics of HIV and other STIs among FSWs are some of the main public health challenges [8], [9], [10]. The percentage positivity of syphilis, CT, NG and HIV were reported to be 8.4%, 14.7%, 5.4% and 0.3% respectively among the FSWs participating in a cross-sectional study using convenience sampling [11]. However, to the best of our knowledge, no study has ever evaluated the correlates of CT and NG infections among FSWs in this province while published evidences regarding current epidemiological situation of syphilis, CT, NG and HIV among FSWs in Jiangsu are also scanty. Thus, all the intervention programs implemented so far among FSWs in this province were based on the experiences from successful strategies followed in other regions. Moreover, the available data regarding these epidemics in Jiangsu till date was mostly based on passive surveillances and thus was very much likely to suffer from miss/under reporting, under-recognition of diseases and huge variation in the quality of reporting systems [12]. Paucity of evidences regarding the HIV/STI burden and their correlates among FSWs of Jiangsu thus called for a comprehensive survey to understand the dynamics of HIV and STI epidemic in this population in order to identify the gaps and guide the policy-makers in designing intervention programs specifically targeted towards bridging them to control these epidemics among FSWs in Jiangsu.

Hence the objectives of this study were to estimate the prevalence of HIV and STIs among FSWs in the Jiangsu Province and to measure the association of CT and NG infections with their potential correlates.

## Methods

### Recruitment

This cross-sectional study was conducted in two cities (Yangzhou and Changzhou) in the Jiangsu province, between June and September 2009 as a part of the baseline survey for a huge integrated project (Mega Project). The Mega Projects were conducted by the China Ministry of Science and Technology (MOST) and Ministry of Health (MOH) to address the most important public health issues in China. The objective of this Mega Project was to identify and establish cohorts of high-HIV risk subpopulations (including FSWs) for the evaluation of the impact of expanded STI care on new HIV infection rate among those high-risk groups. In this project, to recruit a representative sample of FSWs, commercial sex work sites/venues were mapped and categorized according to the average socio-economic condition of the clients visiting each study site [3], [13].

Based on the available information regarding prevalence of syphilis (4.4% in 2008) among FSWs in Jiangsu [14], required sample size was calculated to be1616, assuming an α level (two-sided) of 0.05 and a relative precision of 1%. Following the recommendations of Furstenberg et al [15], to have a sample with stable variance by recruiting at least 5 FSWs from each commercial sex-work venue, 340 venues (targeting a sample size of 1700) were required to be selected (at least 170 in each city). Exhaustive lists of venues were thus prepared for both the cities. Following the methods used by other contemporary researchers, the venues where FSWs usually met their clients were classified into three subgroups: high, middle and low-level venues [3]. High-level venues referred to karaoke bars and hotels, middle-level included hair salons/barber shops, massage parlors, foot-bathing shops, roadside shops, guesthouses and roadside restaurants while low-level consisted of streets and other public outdoor places. To have a representative sample, with the help of stratified random selection procedure for selecting venues using probability proportional to size sampling, 185 venues (39 high, 99 middle and 47 low-level) in Yangzhou and 174 venues (35 high, 92 middle and 47 low-level) in Changzhou were selected. FSWs present at the time of survey in each of the selected sites were recruited if they met the eligibility criteria. To meet the inclusion criteria, the participants had to be: (1) biologically female; (2) involved in providing commercial sex in sex-work venues or rented apartments for money or goods during the previous three months; (3) aged 15 years or more and (4) willing to participate by providing informed consent. Participants who met the following criteria were excluded: (1) medical reasons or intoxication preventing from active participation; (2) currently or previously (during past 3 months) enrolled in any HIV behavioral intervention trial. All potential participants who declined to participate or otherwise did not participate were eligible for treatment and were not disadvantaged in any other way by not participating in the study.

### Structured Interview

After the assessment of eligibility, written informed consents were collected from eligible subjects regarding participation in the study, collection of blood for free HIV and syphilis testing and cervical swab for free NG and CT testing.

A face-to-face interview using an interviewer-administered, pre-tested, structured questionnaire was conducted for each participant to collect information on demographics and recent sexual behaviors.

The demographic information included age (less than 20/20–29/30–39/40 and above), ethnicity (Han/others), education level (illiterate/elementary school/junior high school/senior high school/college and higher), marital status (never married/married/divorced or widowed), living status (with none/regular partner/causal partner/family members/others) and residency [official residency (Hukou) of the cities under study/Jiangsu province/other provinces].

Recent sexual behaviors were assessed by collecting information (yes/no) on: condom use during the last intercourse with clients and unprotected vaginal intercourse (UVI) which was defined as non-consistent use of condom during the previous three months while being engaged in commercial vaginal intercourse.

### Laboratory Tests

Five ml of venous blood was collected from each participant for HIV and syphilis testing. HIV antibodies were screened using a rapid test (Acon Biotech Co., Ltd) and positive samples were re-tested by ELISA (Livzon Pharmaceutical Group Co., Guangzhou, China). Blood samples positive for both tests were then subjected to Western Blot (HIVBLOT 2.2, Genelabs Diagnostics, Singapore) for confirmation of diagnosis. Syphilis antibodies were screened using ELISA (Wantai Biopharmacy Co., Ltd) and positive results were confirmed with Toluidine Red Unheated Serum Test [TRUST: A Qualitative and Quantitative Card Test for the Serologic Detection of Syphilis (Wantai Biopharmacy Co., Ltd)]. Western Blot positive participants were defined as HIV positive while persons positive for both ELISA and TRUST were defined as Syphilis positive.

The approximate sample size required for CT was 918 (using available information for Jiangsu) [14] and for NG it was 731 (using parameters from a contemporary study [16] in another province as for Jiangsu it was not available), assuming an α level (two-sided) of 0.05 and a relative precision of 2%. Thus to enhance the cost-effectiveness while maintaining an average sample size we planned to select a random subsample of all recruited subjects, using blocking (through randomized block numbers equal for both cities) to have a comparable number for both cities through balanced recruitment and 849 subjects were selected in the process (414 in Yangzhou and 435 in Changzhou). Cervical specimens of the selected subjects were evaluated at the National STD Reference Laboratory at Nanjing for NG and CT testing by using Polymerase Chain Reaction (PCR: Roche Amplicor assay, Roche Diagnostic Systems, Indianapolis, IN).

Participants who were HIV positive were referred to the National HIV Care and Treatment Program and STI (syphilis, CT or NG) positives were referred to designated clinics or disease control centers for counseling, treatment and follow up.

### Data Analysis

Data was double-entered using the software EpiData 3.0 [17] and multiple logic checks were used to ensure the data quality. SAS version 9.1 [18] was used for all statistical analyses. Descriptive analyses were conducted to determine the distribution of the demographic factors, sexual behaviors and to calculate the prevalence proportions [with 95% confidence intervals (95%CI)] of HIV and other STIs. In addition, to assess the strength and direction of the association between CT/NG infection and their potential correlates, simple logistic regressions were performed for univariate analysis [Odds ratio (OR) and 95%CI] and stepwise backward model selection method was used next for the multivariate analysis (variables having OR for at least one category with a p-value of less than 0.2 were included in the adjusted model) using multiple logistic regression.

### Ethics Statement

The study process and content, as well as the consent procedures, were approved by the Ethics Committee of Jiangsu Provincial Center for Disease Prevention and Control (JSCDC). Signed informed consent was obtained from each participant prior to the interviews, blood collection and cervical swab collection. The ethics committee were notified that some participants were aged 15 to 18; consent from a parent or guardian was not possible.

## Results

### Demographics and sexual behaviors

Overall 2972 FSWs were recruited, 1108 (37.3%) from Yangzhou and 1864 (62.7%) from Changzhou. Number of participants recruited from high, middle and low-level venues in Yangzhou were: 425 (38.4%), 393 (35.4%) and 290 (26.2%), and in Changzhou were: 735 (39.4%), 982 (52.7%) and 147 (7.9%). About half of the participants (53.3%) were aged between 20 to 29 years and majority (98.5%) belonged to the Han race. Approximately 80% attended junior high school or less, 50% were currently married, 29% were living with their regular partners while another 9% were living with casual partners. Only about 22% were official residents of the cities under study and approximately 46% used to meet their clients at the middle-level commercial sex venues. About 18.5% of the participating FSWs reported that they did not use condom during the last sex with client and about 44% were engaged in UVI. Ever use of any kind of illicit drug in the past year was reported by only 0.37% of the participants. (Table 1)

**Table 1:** Demographics, sexual behavior and syphilis prevalence among recruited FSWs in the Yangzhou and Changzhou cities of Jiangsu, China (N = 2972).

Variables	Categories	Tested for CT/NG			Not tested for CT/NG			Overall	
		n	%	95%CI	n	%	95%CI	n	%
Age	Less than 20	107	12.63	10.39,14.87	316	14.92	13.40,16.43	423	14.27
	20–29	454	53.60	50.24,56.97	1125	53.10	50.99,55.24	1579	53.25
	30–39	238	28.10	25.07,31.13	540	25.50	23.64,27.35	778	26.24
	40 and above	48	5.67	4.11,7.23	137	6.47	5.42,7.52	185	6.24
Ethnicity	Han	822	97.97	97.02,98.93	2072	98.67	98.18,99.16	2894	98.47
	Others	17	2.03	1.07,2.98	28	1.33	0.84,1.82	45	1.53
Education	Illiterate	8	0.94	0.29,1.60	36	1.70	1.15,2.25	44	1.48
	Elementary school	103	12.15	9.94,14.35	317	14.98	13.46,16.50	420	14.17
	Junior High school	592	69.81	66.72,72.90	1295	61.20	59.12,63.28	1887	63.66
	Senior high school	133	15.68	13.23,18.14	429	20.27	18.56,21.99	562	18.96
	College and higher	12	1.42	0.62,2.21	39	1.84	1.27,2.42	51	1.72
Marital status	Never Married	382	45.53	42.15,48.91	1000	47.33	45.20,49.46	1382	46.82
	Married	434	51.73	48.34,55.12	1052	49.79	47.65,51.92	1486	50.34
	Divorced	20	2.38	1.35,3.42	58	2.74	2.05,344	78	2.64
	Widowed	3	0.36	0.00,0.76	3	0.14	0.00,0.30	6	0.20
Living with	None	259	30.80	27.67,33.92	688	32.90	30.89,34.92	947	32.30
	Regular partner	263	31.27	28.13,34.41	599	28.65	26.71,30.59	862	29.40
	Casual partner	79	9.39	7.42,11.37	177	8.46	7.27,9.66	256	8.73
	Family members	29	3.45	2.21,4.68	110	5.26	4.30,6.22	139	4.74
	Others	211	25.09	22.15,28.02	517	24.73	22.87,26.58	728	24.83
Resident of (Hukou)	Cities under study	190	22.51	19.69,25.34	452	21.29	19.55,23.03	642	21.64
	Jiangsu Province	160	18.96	16.31,21.61	476	22.42	20.64,21.20	636	21.44
	Other Provinces	494	58.53	55.20,61.86	1195	56.29	54.18,58.40	1689	56.93
City	Yangzhou	414	37.36	34.51,40.22	694	62.64	59.78,65.49	1108	37.28
	Changzhou	435	23.34	21.42,25.26	1429	76.66	74.74,78.58	1864	62.72
Venue types	High level	346	40.75	37.44,44.96	814	38.34	36.27,40.41	1160	39.03
	Middle level	369	43.46	46.80,49.12	1006	47.39	45.26,49.51	1375	46.27
	Low level	134	15.78	13.33,18.24	303	14.27	12.78,15.76	437	14.70
During last sex with client	Condom used	620	81.79	79.04,84.55	1527	81.44	79.68,83.20	2147	81.54
	Condom not used	138	18.21	15.45,20.96	348	18.56	16.80,20.32	486	18.46
Used any kind of illicit drug in the past year	Yes	5	0.59	0.07,1.11	6	0.28	0.06,0.51	11	0.37
	No	840	99.41	98.89,99.03	2112	99.72	99.49,99.94	2952	99.63
UVI	Yes	320	43.53	39.94,47.13	804	43.67	42.27,46.97	1124	44.34
	No	415	56.46	52.87,60.06	996	55.33	53.03,57.63	1411	55.66
Syphilis	Negative	805	94.82	93.32,96.31	2022	95.24	94.33,96.15	2827	95.12
	Positive	44	5.18	3.69,6.68	101	4.76	3.85,5.66	145	4.88

N: Total number of recruited FSWs.

n: Number of FSWs in each subcategories.

### HIV and STIs Prevalence

In this study, 145 FSWs were positive for syphilis, with an overall prevalence of 4.88% (2.80% in Yangzhou and 6.12% in Changzhou) in the study sample (Table 1 and 2). Six HIV positive FSWs were found in Changzhou (0.32%) and none in Yangzhou, thus constituting an overall HIV percentage positivity of 0.20% among participants. (Table 2)

**Table 2:** Prevalence of HIV, Syphilis, CT and NG among FSWs in Jiangsu, China.

Disease		Yangzhou			Changzhou			Overall		
		N	%	95%CI	n	%	95%CI	n	%	95%CI
HIV(N = 2972)	Positive	0	0.00	–	6	0.32	0.13, 0.74	6	0.20	0.08, 0.46
	Negative	1108	100.00	–	1858	99.68	99.26, 99.87	2966	99.80	99.54, 99.92
Syphilis(N = 2972)	Positive	31	2.80	1.82, 3.77	114	6.12	5.03, 7.20	145	4.88	4.15, 5.73
	Negative	1077	97.20	96.23, 98.17	1750	93.88	92.80, 94.97	2827	95.12	94.27, 95.85
CT(N = 849)	Positive	55	13.29	10.00, 16.57	69	15.86	12.42, 19.31	124	14.61	12.33, 17.20
	Negative	359	86.71	83.43, 90.00	366	84.14	80.69, 87.58	725	85.39	82.80, 87.67
NG(N = 849)	Positive	36	8.70	5.97, 11.42	10	2.30	0.88, 3.71	46	5.42	4.04, 7.22
	Negative	377	91.30	88.58, 94.03	425	97.70	96.29, 99.12	803	94.58	92.78, 95.96

N: Total number of FSWs tested for the corresponding diseases.

n: Number of FSWs in each subcategories.

Participants who were selected for CT and NG testing were not much different in terms of demographic and behavioral factors from those who didn't get tested as evident from the overlapping 95%CIs presented in Table 1. The overall CT prevalence among these 849 recruited FSWs was 14.61% while the prevalence in Yangzhou and Changzhou were 13.29% and 15.86% respectively. On the other hand the overall NG prevalence in the sample was 5.42% while the prevalence in Yangzhou and Changzhou were 8.70% and 2.30% respectively (Table 2).

### Correlates of CT infection

Both univariate and multivariate analyses indicated that compared to the 20–29 years age group, recruited FSWs aged 30–39 years were less likely to suffer from CT infection (unadjusted Odds Ratio: OR = 0.49, 95%CI: 0.30–0.82 while adjusted Odds Ratio: AOR = 0.52, 95%CI: 0.27–0.98). The youngest age group (aged less than 20 years) seemed to have highest odds of CT acquisition (OR = 1.39, 95%CI: 0.82–2.35; AOR = 1.58, 95%CI: 0.82–3.06) although the result lacked statistical power. Compared to those who attended junior high school, participants who attended senior high school (OR = 0.42, 95%CI: 0.27–0.67; AOR = 0.40, 95%CI: 0.23–0.70) had lower odds of having CT infection while FSWs having elementary school level education did show a lower likelihood of being CT positive (OR = 0.48, 95%CI: 0.24–0.96) in the unadjusted model. Univariate analysis also found that FSWs living with regular partners were less likely to acquire CT (OR = 0.50, 95%CI: 0.30–0.84) while those living with others (other than partners or family members) had much higher odds of being CT positive (OR = 2.40, 95%CI: 1.05–5.49) compared to those living alone. Recruited FSWs who were not the official residents of the cities under study, tended to have higher chances of CT infection (OR = 1.61, 95%CI: 0.87–2.98; AOR = 1.54, 95%CI: 0.73–3.28 for the residents of other cities in Jiangsu and OR = 1.53, 95%CI: 0.92–2.56; AOR = 1.25, 95%CI: 0.64–2.45 for the residents of other provinces) compared to the official residents of Changzhou and Yangzhou but these results also lacked power. (Table 3)

**Table 3:** Associations of demographic factors, sexual behavior and other STIs with CT infection among selected FSWs in the Yangzhou and Changzhou cities of Jiangsu, China (N = 849).

Variables		Unadjusted		Adjusted	
		OR(95%CI)	p value	OR(95%CI)	P value
Age	Less than 20	1.39(0.82,2.35)	0.22	1.58(0.82,3.06)	0.17
	20–29	Reference		Reference	
	30–39	0.49(0.30,0.82)	0.01*	0.52(0.27,0.98)	0.04*
	40 and above	0.59(0.23,1.54)	0.28	0.62(0.21,1.87)	0.40
Ethnicity	Others	Reference		#	
	Han	0.78(0.18,3.45)	0.74		
Education	Illiterate	1.02(0.20,5.30)	0.98	1.52(0.25,9.17)	0.64
	Elementary school	0.48(0.24,0.96)	0.04*	0.72(0.32,1.60)	0.42
	Junior High school	Reference		Reference	
	Senior high school	0.42(0.27,0.67)	<0.001*	0.40(0.23,0.70)	0.001*
	College and higher	1.02(0.26,3.99)	0.98	0.60(0.11,3.26)	0.55
Marital status	Never Married	Reference		Reference	
	Married	0.70(0.48,1.04)	0.08	1.44(0.73,2.82)	0.29
	Divorced/Widowed	0.54(0.12,2.38)	0.41	0.91(0.17,4.79)	0.91
Living with	None	Reference		Reference	
	Regular partner	0.50(0.30,0.84)	0.01*	0.63(0.33,1.23)	0.18
	Casual partner	0.66(0.32,1.38)	0.27	0.52(0.22,1.24)	0.14
	Family members	0.76(0.46,1.24)	0.27	0.76(0.40,1.48)	0.62
	Others	2.40(1.05,5.49)	0.04*	2.30(0.85,6.22)	0.10
Resident of (Hukou)	Cities under study	Reference		Reference	
	Jiangsu Province	1.61(0.87,2.98)	0.14	1.54(0.73,3.28)	0.26
	Other Provinces	1.53(0.92,2.56)	0.10	1.25(0.64,2.45)	0.51
City	Yangzhou	Reference		#	
	Changzhou	1.23(0.84,1.80)	0.29		
Venue types	High level	Reference		Reference	
	Middle level	1.38(0.91,2.08)	0.13	1.25(0.72,2.17)	0.43
	Low level	0.91(0.49,1.67)	0.75	0.74(0.31,1.76)	0.49
During last sex with client	Condom used	Reference		#	
	Condom not used	1.04(0.62,1.75)	0.88		
UVI	No	Reference		Reference	
	Yes	1.44(0.98,2.11)	0.06	1.78(1.10,2.87)	0.02*
Syphilis	Negative	Reference		Reference	
	Positive	2.04(1.00,4.16)	0.05	1.81(0.83,3.96)	0.14
NG	Negative	Reference		Reference	
	Positive	3.08(1.61,5.89)	<0.001*	2.72(1.29,5.76)	0.01*

N: Total number of FSWs tested for the corresponding disease.

Indicates p-value less than 0.05.

#: Variable not included in the adjusted model (as p values for the unadjusted association for all categories were >0.2).

Being engaged in UVI was associated with higher odds of acquiring CT (AOR = 1.78, 95%CI: 1.10–2.87) after adjusting for other variables while similar indication was also there in the unadjusted analysis that lacked power (OR = 1.44, 95%CI: 0.98–2.11). Having syphilis seemed to be positively associated with having CT (OR = 2.04, 95%CI: 1.00–4.16; AOR = 1.81, 95%CI: 0.83–3.96) while being positive for NG was strongly associated with CT infection (OR = 3.08, 95%CI: 1.61–5.89; AOR = 2.72, 95%CI: 1.29–5.76) in both unadjusted and adjusted models.

### Correlates of NG infection

Han ethnicity (OR = 2.42, 95%CI: 0.54–10.90; ref: others), college or higher level of education (OR = 3.63, 95%CI: 0.66–19.83; AOR = 7.15, 95%CI: 0.88–58.39; ref: senior high school level), being married (OR = 1.73, 95%CI: 0.91–3.30; AOR = 1.84, 95%CI: 0.75–4.53; ref: never married) and working in low-level sex-work venues (OR = 2.19, 95%CI: 0.89–5.24; AOR = 2.26, 95%CI: 0.77–6.62; ref: high-level) seemed to indicate higher likelihood of having NG but neither results were statistically significant. Univariate analyses revealed that, divorced/widowed FSWs (OR = 4.44, 95%CI: 1.17–16.79) and those who were selected from middle-level sex-work venues (OR = 2.31, 95%CI: 1.12–4.75; ref: high-level) had higher odds of being infected with NG. Subjects living with family members (OR = 0.34, 95%CI: 0.14–0.87; AOR = 0.35, 95%CI: 0.13–0.93; ref: living alone) and working in venues at Changzhou (OR = 0.25, 95%CI: 0.12–0.50; AOR = 0.13, 95%CI: 0.06–0.30; ref: Yangzhou) were less likely to be NG positive in both unadjusted and adjusted analyses. Engaging in UVI (OR = 2.05, 95%CI: 1.13–3.73; AOR = 1.96, 95%CI: 0.92–4.18) and being positive for Syphilis (OR = 3.02, 95%CI: 1.21–7.56; AOR = 4.70, 95%CI: 1.58–13.98) or CT (OR = 3.08, 95%CI: 1.61–5.89; AOR = 2.76, 95%CI: 1.32–5.81) were associated with higher likelihood of NG positivity (Table 4).

**Table 4:** Association of demographic factors, sexual behavior and other STIs with NG infection among selected FSWs in the Yangzhou and Changzhou cities of Jiangsu, China (N = 849).

Variables		Unadjusted		Adjusted	
		OR(95%CI)	p value	OR(95%CI)	p value
Age	Less than 20	1.21(0.51,2.87)	0.67	#	
	20–29	Reference			
	30–39	1.00(0.50,1.98)	0.99		
	40 and above	0.37(0.05,2.77)	0.33		
Ethnicity	Others	Reference		#	
	Han	2.42(0.54,10.90)	0.25		
Education	Illiterate	-		-	
	Elementary school	1.12(0.36,3.45)	0.84	0.58(0.17,1.97)	0.38
	Junior High school	1.00(0.43,2.33)	1.00	0.67(0.26,1.71)	0.40
	Senior high school	Reference		Reference	
	College and higher	3.63(0.66,19.83)	0.14	7.15(0.88,58.39)	0.07
Marital status	Never Married	Reference		Reference	
	Married	1.73(0.91,3.30)	0.09	1.84(0.75,4.53)	0.25
	Divorced/Widowed	4.44(1.17,16.79)	0.03*	2.55(0.43,15.14)	0.30
Living with	None	Reference		Reference	
	Regular partner	0.61(0.30,1.24)	0.17	1.06(0.47,2.43)	0.88
	Casual partner	0.46(0.13,1.59)	0.22	0.76(0.20,2.85)	0.69
	Family members	0.34(0.14,0.87)	0.02*	0.35(0.13,0.93)	0.04*
	Others	1.35(0.38,4.84)	0.64	1.63(0.38,6.97)	0.51
Resident of (Hukou)	Cities under study	Reference		#	
	Jiangsu Province	0.76(0.29,2.02)	0.59		
	Other Provinces	1.00(0.49,2.06)	0.99		
City	Yangzhou	Reference		Reference	
	Changzhou	0.25(0.12,0.50)	<0.001*	0.13(0.06,0.30)	<0.001*
Venue types	High level	Reference		Reference	
	Middle level	2.31(1.12,4.75)	0.02*	2.03(0.83,4.99)	0.12
	Low level	2.19(0.89,5.42)	0.09	2.26(0.77, 6.62)	0.14
During last sex with client	Condom used	Reference		#	
	Condom not used	0.87(0.38,1.99)	0.74		
UVI	No	Reference		Reference	
	Yes	2.05(1.13,3.73)	0.02*	1.96(0.92,4.18)	0.08
Syphilis	Negative	Reference		Reference	
	Positive	3.02(1.21,7.56)	0.02*	4.70(1.58,13.98)	0.005*
CT	Negative	Reference		Reference	
	Positive	3.08(1.61,5.89)	<0.001*	2.76(1.32,5.81)	0.01*

N: Total number of FSWs tested for the corresponding disease.

Indicates p-value less than 0.05.

‘-’Refers to situations where valid ORs couldn't be determined due to inadequate cell values.

#: Variable not included in the adjusted model (as p values for the unadjusted association for all categories were >0.2).

## Discussion

In this comprehensive survey involving a representative sample of FSWs in two cities of Jiangsu province of China, the prevalence of STIs like CT (14.61%), NG (5.42%) syphilis (4.88%) and HIV (0.20%) were measured.

The observed proportions for CT and NG were lower than the findings from studies conducted among FSWs in Kaiyuan city in 2006 and 2008 [16], [19], Guangzhou [20] and Gejiu [21]. Compared to a previous observation in Shenzhen, the prevalence of CT was higher while for NG it was much lower [22]. Syphilis prevalence measured in this study was similar to that in Sichuan province [23], lower than the observations in southwestern China [24]and India [25], but higher than some European countries [26], [27]. Being one of the first investigations on burden of syphilis, CT and NG in Jiangsu based on a representative sample recruited through a multistage sampling frame involving probability sampling of commercial sex work venues, the observed proportions may be considered as realistic pictures of these STI epidemics in this province necessitating urgent target-oriented disease control programs among FSWs. While the differences in the observed burden might have resulted from differences in sampling techniques across the studies, the probability of having different epidemic situations in the source population of FSWs in respective areas should also be borne in mind.

Although only six FSWs were found to be positive for HIV in this study, keeping in mind the fact that the risk of HIV acquisition increases manifold in presence of STIs like CT, NG and syphilis [28], [29], [30], considerable number of identified cases of these STIs among FSWs in Jiangsu probably indicated towards the worrisome potential for inducing upsurge of HIV epidemic in this population. Additionally, by increasing immune activation of host cells and secretion of cytokines, these STIs may enhance viral replication among HIV patients, resulting in accelerated progression to AIDS [30].

Recruited FSWs were mostly young (93.8% were aged less than 40 years) and educated up to high-school level (only 1.7% had college-level or higher education). Lack of awareness may translate this lower educational attainment into increased risk of acquisition of STIs including HIV [31] due to greater likelihood of engaging in risky sexual behaviors [32], [33]. Potential for being engaged in high-risk sexual behaviors might also be high in this population as 46.8% of them were never married and 34.1% were living with regular partners or family members [34]. A previous study evidenced that FSWs in China are highly mobile with turnover rate across venues being once in 3–4 months [4]. In our study, only 21.6% FSWs were the official residents of the city from where they were recruited, probably indicating that majority of this population was migrated and thus more likely to have risky sexual behaviors. Hence roll out of disease control programs to them seemed difficult [35]. 81.5% of the participants used condom during their last sex with clients but 44.3% did not use condom consistently for the past three months. Considering the evidences from contemporary scientific literature, these demographic and sexual behavioral patterns among FSWs of Jiangsu were likely to emphasize the vulnerability of this population regarding acquisition of HIV and other STIs [36], [37].

While estimating the magnitude and direction of association between CT/NG infections and their potential correlates, younger FSWs seemed to be more vulnerable for CT acquisition. This finding corroborated with observations from other studies [3], [19] and might be explained by the potential lack of awareness and access regarding STI control programs among younger FSWs along with their increased risk for getting exposed to other possible contributors like sexual violence and increased number of sexual acts. We didn't find any consistent pattern of association between education and risk of acquiring CT or NG. Compared to the FSWs who were never married, divorced or widowed were much more likely to have NG infection. Similar observation had also been reported in other countries [38]. Lack of social and economic support might have compelled divorced or widowed women of lower socio-economic tier to get involved in this trade and exposed to sexual violence resulting in higher risk of STI acquisition. Social isolation and sexual deprivation might also be the reasons behind their vulnerability for unprotected sex and high risk sexual behaviors. These explanations may also support our observation that FSWs living with family members, regular or casual partners were less likely to acquire CT or NG compared to those who lived alone or with others.

Participants who used to work at middle or low-level commercial sex work venues had much higher risk of contracting NG infection compared to those in high-level venues. These findings corroborated with observations from several other studies [3], [19], [24], [39]. FSWs in the middle or lower-level venues probably had poor awareness and less social safety, hence were more vulnerable to sexual violence, unprotected sex and risky behaviors including serving more clients compared to their counterparts in high-level venues [40].

Official residency of other cities/provinces seemed to be associated with higher risk of CT infection compared to the residents of Yangzhou and Changzhou, among FSWs sampled from these two cities. Thus migrating from other areas for sex work was probably associated with increased risk of STI acquisition in this population [34], which may well result in worsening of the HIV and STI epidemic situations in Jiangsu Province.

Alike others [16], [20], our results also indicated that UVI was an important risk factor for acquisition of CT or NG among FSWs. These findings probably emphasized on the urgent need for condom promotion in commercial sex work venues of Yangzhou and Changzhou.

In the current study sample, having other STI (syphilis, NG for CT or CT for NG) was positively associated with risk of acquiring each of these diseases. Similar results were reported in several other studies conducted among FSWs [19]. As all these infections share the similar mechanisms of transmission, infection by one organism offers more potential opportunities for others [3], [19].

According to our knowledge this was the first effort in Jiangsu to determine the association of CT and NG infections with their potential predictors. By virtue of its sampling design this study was able to recruit a representative population of FSWs in two cities of Jiangsu. The measured prevalence of HIV and other STIs as well as the observed associations of CT and NG infections with their potential predictor can thus be extrapolated by the policy-makers for the purpose of designing appropriate targeted interventions. Large sample size, use of biological markers, advanced laboratory investigation techniques and following uniform study protocol for extensive training of all study personnel to minimize interviewer bias were major strengths of this study. Moreover, prompt referral of the FSWs diagnosed with HIV/syphilis/CT/NG to the appropriate treatment/counseling center for proper treatment and follow up can also be considered as an additional benefit from this study.

There were quite a few limitations in our study. Because of the cross-sectional design, temporal ambiguity prevented us from drawing causal inferences based on our results and we recommend that any such interpretation should be made with caution. Vulnerability of the self-reported information to social desirability bias, might lead to misclassification in our study. But we think even if misclassifications were there they were most likely to be non-differential as the STI positivity status of the subjects were not determined at the time of the interview. Although there remained some potential for differential misclassification due to the possibility of the responses by FSWs having symptoms or prior diagnosis of any STI being different from others, we still believe that the magnitude will be negligible. Selection bias and lack of generalizability were other likely shortcomings but they were probably been taken care of by our sampling technique which was planned for recruiting a representative population. Regarding the selection of venues of commercial sex work, although we used probability sampling (probability proportional to size with stratified random sampling), in the next stage we used convenience sampling (all the FSWs present in the venue were recruited). Hence the chances of over-representation of more sexually active FSWs were always there, but we believe that sexual activity of the recruited subjects was less likely to be an important biasing factor. Despite of having a larger overall sample, the size of chosen subsample for CT/NG testing was not sufficient enough for having adequate power of analysis while determining the association of some of the demographic and behavioral factors with CT and NG. As it was not possible for us to have an exhaustive questionnaire while interviewing the FSWs in the sex-work venues, information was collected only on selected sexual behaviors and covariates leading to the possibilities of residual confounding.

### Conclusion

Based on the representative sample, despite of all the limitations, it can be concluded that substantial number of STI cases were found to be present among FSWs in Yangzhou and Changzhou and having any STI was highly correlated with the risk of having others. Focused interventions are urgently required for controlling the epidemics of HIV and other STIs among FSWs of Jiangsu province of China, specifically targeting those who are young, divorced/widowed, living alone or with persons other than partners or family members and habituated in unprotected commercial vaginal intercourse.
